# Antimicrobial peptide activity in asymmetric bacterial membrane mimics†

**DOI:** 10.1039/d1fd00039j

**Published:** 2021-07-21

**Authors:** Lisa Marx, Moritz P. K. Frewein, Enrico F. Semeraro, Gerald N. Rechberger, Karl Lohner, Lionel Porcar, Georg Pabst

**Affiliations:** University of Graz, Institute of Molecular Biosciences, NAWI Graz 8010 Graz Austria georg.pabst@uni-graz.at +43 316 380 4989; Field of Excellence BioHealth, University of Graz Graz Austria; Institut Laue-Langevin 38043 Grenoble France

## Abstract

We report on the response of asymmetric lipid membranes composed of palmitoyl oleoyl phosphatidylethanolamine and palmitoyl oleoyl phosphatidylglycerol, to interactions with the frog peptides L18W-PGLa and magainin 2 (MG2a), as well as the lactoferricin derivative LF11-215. In particular we determined the peptide-induced lipid flip-flop, as well as membrane partitioning of L18W-PGLa and LF11-215, and vesicle dye-leakage induced by L18W-PGLa. The ability of L18W-PGLa and MG2a to translocate through the membrane appears to correlate with the observed lipid flip-flop, which occurred at the fastest rate for L18W-PGLa. The higher structural flexibility of LF11-215 in turn allows this peptide to insert into the bilayers without detectable changes of membrane asymmetry. The increased vulnerability of asymmetric membranes to L18W-PGLa in terms of permeability, appears to be a consequence of tension differences between the compositionally distinct leaflets, but not due to increased peptide partitioning.

## Abstract 2

We report on the response of asymmetric lipid membranes composed of palmitoyl oleoyl phosphatidylethanolamine and palmitoyl oleoyl phosphatidylglycerol, to interactions with the frog peptides L18W-PGLa and magainin 2 (MG2a), as well as the lactoferricin derivative LF11-215.

## Graphical abstract

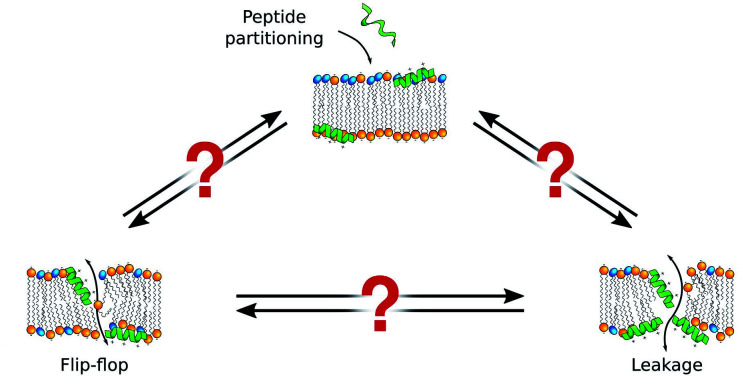

## Introduction

Antimicrobial peptides (AMPs) are widely studied compounds of the innate immune system with high potential to combat the spread of infectious diseases due to multi-resistant strains.^1,2^ Compared to conventional antibiotics, AMPs translocate or impair cellular envelopes *via* unspecific molecular interactions (electrostatic, hydrophobic, entropic), although their final target might also be located in the cytosolic compartment.^3^ Various models have been reported for AMP/lipid interactions, including the formation of transmembrane peptide pores, micellization, or interfacial activity, the latter of which may lead to the formation of a surface-adsorbed peptide layer (carpet) or peptide self-aggregation.^4–6^ Peptide pore formation has also been connected to accelerated lipid flip-flop,^7–9^ and recently confirmed for asymmetric lipid membranes.^10–12^

On the other hand, it is increasingly clear that the mode of action of a given peptide depends strongly on the composition of the lipid bilayer.^13,14^ That is, AMPs cannot be classified as pore-formers, or not without referring to the composition of their target membrane. Importantly, the most abundant phospholipids of bacterial membranes are phosphatidylglycerol (PG), phosphatidylethanolamine (PE) and cardiolipin.^15^ For example, PGLa from the African clawed frog was reported to align differently with respect to the membrane surface, depending on lipid composition, hydration level or peptide concentration.^16,17^

It is not clear whether surface-adsorbed AMPs also lead to enhanced lipid flip-flop. Interestingly, all-atom molecular dynamics simulations have suggested that lipids may co-translocate with peptides through membranes.^18^ However, experimental evidence for such a scenario is currently not available. Moreover, bacterial membranes, including cytoplasmic membranes of Gram-negative bacteria, display transbilayer compositional asymmetry,^19^ whose role in the context of AMP activity is basically unknown.

This prompted us to measure lipid flip-flop in asymmetric large unilamellar vesicles (aLUVs) composed of palmitoyl oleoyl phosphatidylethanolamine (POPE) and palmitoyl oleoyl phosphatidylglycerol (POPG) using L18W-PGLa, as well as magainin 2a (MG2a) and the lactoferricin derivative LF11-215, all of which remain surface bound in PE/PG membranes.^20,21^ In particular, we coupled time resolved small-angle neutron scattering (SANS) measurements of lipid flip-flop to dye-leakage and peptide partitioning using Trp emission spectroscopy. Our results reveal a peptide-concentration dependent loss of membrane asymmetry, which was most expressed for L18W-PGLa, followed by MG2a. LF11-215 in turn caused no detectable lipid flip-flop despite its high partitioning into aLUVs. This suggests that the high structural flexibility of LF11-215 enables the peptide to translocate through asymmetric membranes without noticeable effects on membrane structure. For the two linear peptides, L18W-PGLa and MG2a, lipid flip-flop instead appears to be coupled to their translocation probability. The increased permeability of aLUVS in the presence of L18W-PGLa as compared to symmetric vesicles is not due to increased peptide partitioning, but appears to be dominated by an internal lateral stress imbalance between the two leaflets.

## Materials and methods

### Lipids and peptides

POPE, POPG and palmitoyl-d31 oleoyl phosphatidyglycerol (POPG-d31) were purchased from Avanti Polar Lipids (Alabaster, AL) as powder and used without further purification. L18W-PGLa (GMASKAGAIAGKIAKVAWKAL-NH_2_), MG2a (GIGKFLHSAKKFGKAFVGEIMNS-NH_2_), and LF11-215 (FWRIRIRR-NH_2_) were obtained in lyophilized form (purity > 95%) from PolyPeptide Laboratories (San Diego, CA). ANTS (8-aminonaphthalene-1,3,6-trisulfonic acid, disodium salt) and DPX (*p*-xylene-bis-pyridinium bromide) were purchased from Molecular Probes (Eugene, OR) and HEPES (purity > 99.5%) from Carl Roth (Karlsruhe, Germany). D_2_O was obtained from Euroisotop (Saarbrücken, Germany), methyl-β-cyclodextrin (mβCD), Triton X-100 and all other chemicals (*pro analysis* quality) were from Sigma-Aldrich (Vienna, Austria).

### Sample preparation

aLUVs with an outer leaflet enriched in POPE and an inner leaflet composed of POPG were produced using a previously reported protocol.^22^ In short, outer leaflet lipids of POPG acceptor LUVs (size: ∼ 100 nm), suspended in HBS buffer (10 mM HEPES, 140 mM NaCl, pH 7.4), were exchanged *via* mβCD-mediated lipid transfer for POPE. For SANS experiments POPG-d31 was used instead of POPG and the HBS was replaced by buffer prepared in 100% D_2_O (HBSD); for leakage experiments POPG acceptor LUVs containing ANTS/DPX were prepared as described elsewhere.^21^ Outer leaflet exchange was achieved preparing first donor multilamellar POPE vesicles (MLVs), hydrated in HBS with 20 w/w% sucrose (0.632 M), followed by an incubation with mβCD (35 mM) at 40 °C for 2 h. Donor and acceptor vesicles were then mixed at an acceptor/donor ratio of 1 : 2 (mol/mol) and incubated at 40 °C for another 30 min. Exchange vesicles were separated from donor vesicles, mβCD and sucrose as previously detailed.^22^ Vesicle size of acceptor LUVs and aLUVs was checked by dynamic light scattering (DLS) using a Zetasizer Nano ZSP (Malvern Panalytical, Malvern, UK), affirming the integrity of the produced aLUVs and absence of large donor MLVs.

The achieved lipid exchange was determined by ultra-performance liquid chromatography-tandem mass spectrometry (UPLC-MS) for protiated samples and gas chromatography (GC) for aLUVs containing POPE-d31 [see ESI† for details]. UPLC-MS results revealed an overall POPE/POPG ∼ 1 : 2 mol/mol ratio for aLUVs. aLUVs were converted into scrambled LUVs (ScraLUVs), *i.e.* same lipid composition, but symmetrically distributed between the two leaflets, as detailed in ref. 22. Additionally, we also prepared LUVs composed of POPE/POPG (7 : 3 mol/mol) as outer leaflet mimics (OLM) of our aLUVs. Phospholipid concentrations were determined through the Bartlett phosphate assay.^23^

### Leakage assay

Measurements were performed in quartz cuvettes in 2 mL of iso-osmotic HBS buffer containing 1 mM EDTA on a Cary Eclipse Fluorescence Spectrophotometer (Varian/Agilent Technologies, Palo Alto, CA) as detailed in ref. 21. Achieved leakage after the addition of peptide was derived from1
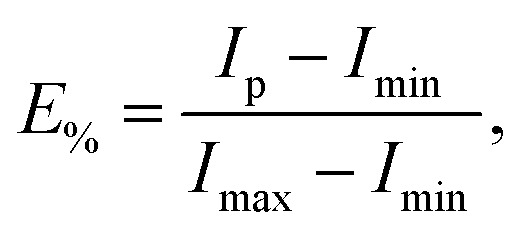
where *I*_min_ is the initial fluorescence without peptide, and *I*_max_ is the fluorescence corresponding to 100% leakage determined through the addition of a 1 vol% solution of Triton X-100.

### Trp-fluorescence spectroscopy

Peptide partitioning was determined from Trp-fluorescence emission for L18W-PGLa and LF11-215 using a Cary Eclipse Fluorescence Spectrophotometer (Varian/Agilent Technologies) at an excitation wavelength of *λ* = 280 nm, and slit widths for incident and outgoing beams of either 5 or 10 nm, as detailed previously.^24^ All samples were contained in a quartz cuvette with a magnetic stirrer and measured at 37 °C. Spectra were analyzed with a linear combination of two independent bands each fitted by a log-normal-like function.^25,26^ This allowed us to extract the molar concentration of dissociated peptide, [P]_W_, and subsequently the molar concentration of membrane-associated peptide [P]_B_ = [P] − [P]_W_, where [P] is the total peptide concentration in the sample.^24^

The mole fraction partitioning coefficient2
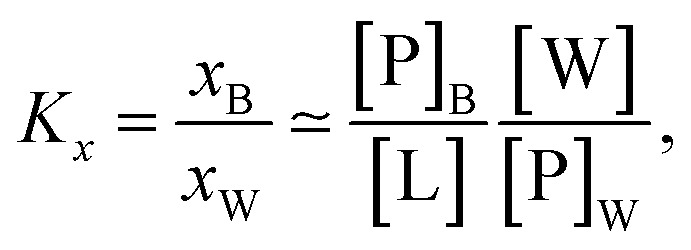
was then calculated for a given lipid concentration [L], where *x*_B_ is the mole fraction of membrane-partitioned peptide, *x*_W_ is the mole fraction of unbound peptide and [W] is the concentration of bulk water (55.3 M at 37 °C).^27,28^ Additionally, we derived the ratio of bound peptide using3
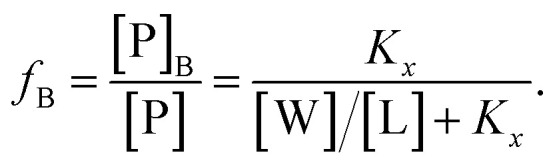


### Small-angle neutron scattering

SANS measurements were performed at the D22-large dynamic range small-angle diffractometer, located at the Institut Laue-Langevin in Grenoble, France, with a two-^3^He-detector setup at a wavelength of 6 Å (*λ*/Δ*λ* = 10%), resulting in a *q*-range of 0.016–0.6 Å^−1^. Flip-flop kinetics were measured with a time resolution of 2 min and sample-to-detector distances (SDD) of 1.3 and 5.6 m, and a 5.6 m collimation; low-*q* measurements of reference (aLUVs) and endstate measurements were conducted at SDDs of 1.3 and 17.8 m, with a 17.6 m collimation. Samples (concentration 7 mg mL^−1^ in HBSD) were measured and filled in Hellma 120-QS cuvettes of 1 mm pathway and equilibrated at 37 °C using a circulating water bath. Data, available at (DOI: 10.5291/ILL-DATA.DIR-217) were reduced using the GRASP-software, performing flat field, solid angle, dead time and transmission corrections, and were normalized by incident flux. Finally, contributions from the empty cell and solvent were subtracted. Data were averaged over 5 to 10 frames to achieve sufficient signal to noise ratios.

**Fig. 1:**
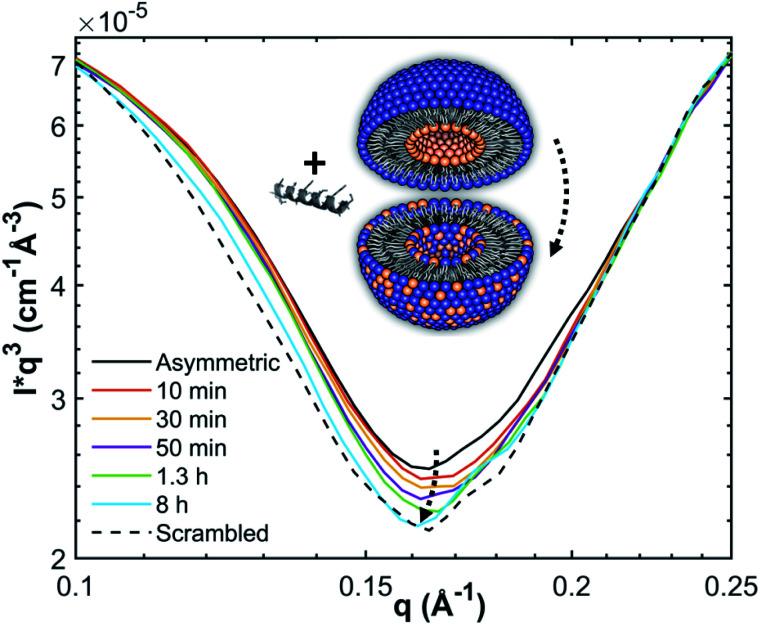
Measurement principle and scattering contrast between (POPG-d31)^in^/(POPE/POPG-d31)^out^ aLUVs and ScraLUVs in HBSD buffer, as observed by SANS at 37 °C. Scattering contrast was additionally enhanced by multiplying the scattered intensities with *q*^3^.

Analogously to ref. 11, lipid flip-flop was determined by measuring the peptide-induced change of scattering contrast with time (Fig. 1). Here, the contrast emerges from chain deuterated POPG-d31, which is primarily located in the inner leaflet, and fully protiated POPE, enriched in the outer leaflet. Then, the change of contrast follows^11,29^4
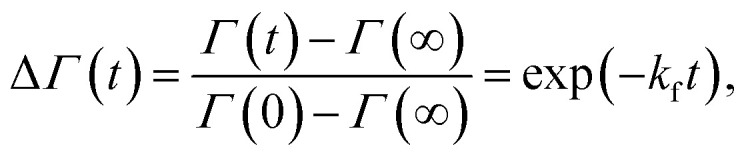
where *Γ* = ∫*Iq*^3^d*q* in the shown *q*-range (Fig. 1), *Γ*(0) corresponds to the initial aLUVs, *Γ*(∞) to ScraLUVs, and *k*_f_ is the lipid flip-flop rate.

## Results

### Asymmetric membranes are more vulnerable to peptide-induced dye efflux

**Fig. 2:**
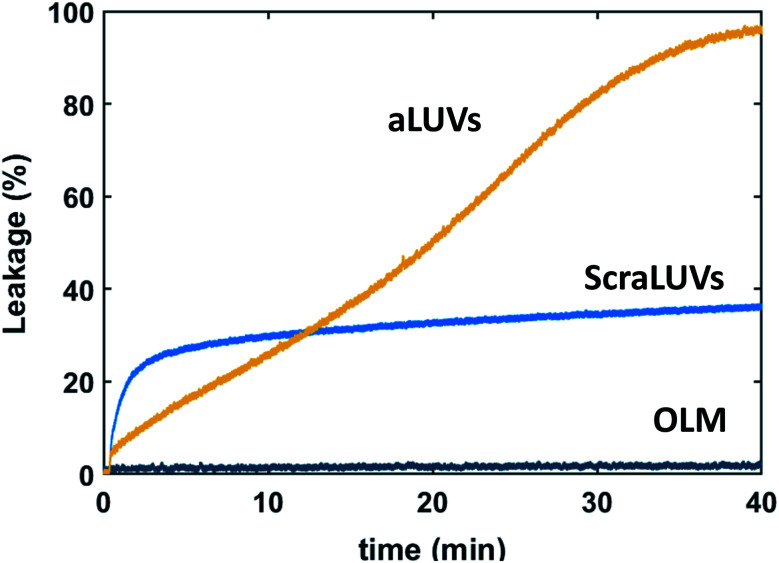
Kinetics of L18W-PGLa-induced dye efflux from (POPG)^in^/(POPE/POPG)^out^ aLUVs, ScraLUVs and OLM for [P]/[L] = 1 : 400 ([L] = 50 μM, *T* = 37 °C).

We started our experiments by studying the kinetics of dye release induced by L18W-PGLa. Fig. 2 shows the observed permeation of aLUVs, ScraLUVs and OLM over a time of 40 min. Symmetric LUVs, mimicking the outer leaflet of our aLUVs were basically impermeable to dyes in the presence of peptides, while ScraLUVs showed initially the fastest leakage increase, but levelled off at ∼38% leakage at the end of the experiment. Instead, dye-efflux for POPE/POPG aLUVs started more gradually, but then reached final leakage values close to 100%. Changing the peptide concentration affected the leakage kinetics most (speeding-up for increasing, and slowing-down for decreasing L18W-PGLa content); a similar effect was observed upon increasing lipid concentration (Fig. S1†).

### AMP partitioning depends on transbilayer lipid distribution

**Fig. 3:**
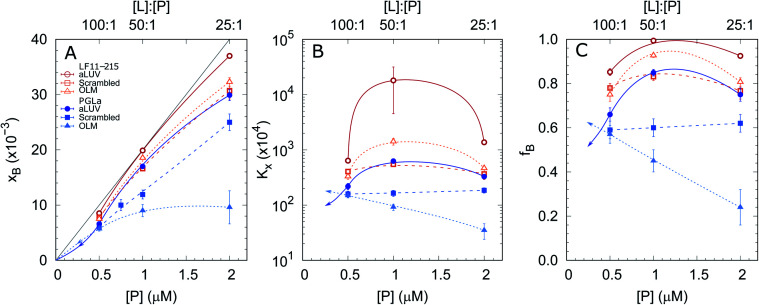
Mole fraction of membrane-partitioned peptides (A), partitioning coefficient (B) and ratio of partitioned peptides (C) as a function of total LF11-215 (open symbols) and L18W-PGLa (filled symbols) concentrations and [L] = 50 μM. Data refer to aLUVs (circles), ScraLUVs (squares) and OLM (triangles). The gray line in (A) represents the limit [P]/[L] corresponding to *f*_B_ = 1; all other lines are guides for the eye. In the case of L18W-PGLa, the arrows indicate a realistic propagation for [P] < 0.5 μM.

In order to shed some light on the increased leakage of aLUVs we first performed peptide partitioning studies making use of the Trp-residue of L18W-PGLa. Further, we included LF11-215, which also contains a Trp-residue. Fig. 3 displays the mole fraction of the partitioned peptides, partitioning coefficient, and ratio of partitioned to total number of peptides as a function of peptide concentration for [L] = 50 μM. All presented data have been taken after 60 min of incubation with the peptides. We also performed time-resolved measurements with the shortest time-interval being ∼20 s after mixing, but observed no noticeable differences to the data recorded after extended incubation times.

Both peptides exhibited the highest affinity to aLUVs, followed by ScraLUVs and OLM in the studied peptide range ([P] = 0.5–2 μM). Moreover, both *K*_*x*_ and *f*_B_ peaked at [P] ∼ 1 μM ([P]/[L] = 1 : 50), and in particular for LF11-215, where *f*_B_ ≃ 1. Similar behavior, but much less pronounced was also observed for symmetric LUVs in the case of LF11-215. In turn *K*_*x*_ remained constant for scrambled LUVs in the presence of L18W-PGLa, mirrored also in a linear increase of *x*_B_ with [P]. In OLM, *K*_*x*_ and *f*_B_ decreased upon increasing L18W-PGLa concentration instead. While the non-monotonous variation of peptide partitioning might indicate a combination of cooperative (increasing *K*_*x*_) and anticooperative (decreasing *K*_*x*_) peptide/peptide or peptide/lipid interactions,^27^ it is interesting that LF11-215 partitions more favorably into OLM than into ScraLUVs at [P] = 1 μM; a situation which is reversed for L18W-PGLa. That is, L18W-PGLa more favorably interacts with ScraLUVs than with OLM.

Our experimental set-up did not allow us to measure peptide concentrations as low as those used for leakage experiments. However, extrapolating roughly the trends observed at lower peptide concentrations ([P] → 0 ⇔ *x*_B_ → 0) suggests that L18W-PGLa partitions less into aLUVs than into both symmetric LUVs under experimental conditions used for the leakage measurements shown in Fig. 2. In order to measure peptide partitioning at [P]/[L] = 1 : 400 we increased the lipid concentration to 200 μM (Fig. S2†). Although increasing lipid concentration is known to affect peptide partitioning,^24,27^ these data support the idea that L18W-PGLa does not preferentially partition into aLUVs under leakage conditions.

### Lipid flip-flop in aLUVs is highly peptide specific

Finally, we determined the peptide-induced lipid flip-flop using SANS combined with a H/D contrast variation scheme that allowed us to discriminate for transbilayer lipid distribution. In particular, we substituted POPG by chain-perdeuterated POPG-d31 in our aLUV preparations and monitored its equilibration across both lipid leaflets by time-resolved SANS as detailed in the Materials and methods section. The scattering patterns of aLUVs and ScraLUVs were typical for single-shelled vesicles and were analysed in terms of a modified 4-slab model^30^ to determine the leaflet composition (Fig. S4†). Specific care was taken to keep the peptide concentrations well below the thresholds reported for POPE/POPG (3 : 1 mol/mol) mixtures for the formation of vesicle aggregation or multilamellar vesicles.^24,31,32^ This was additionally checked by inspection of the final SANS patterns after peptide addition, which did not show any signatures for changes in overall vesicle morphology or aggregation (Fig. S4†).

**Fig. 4:**
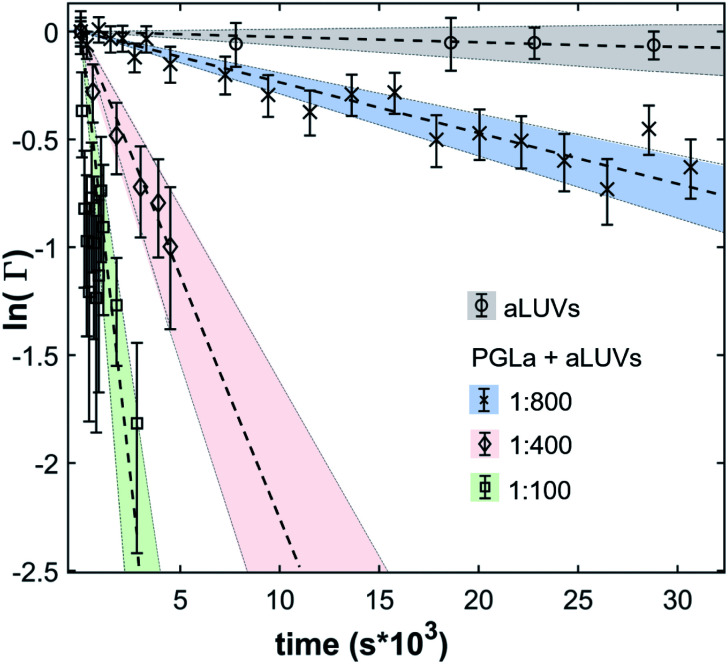
Decay of scattering contrast between aLUVs and scrambled LUVs due to L18W-PGLa-mediated lipid flip-flop at [L] = 9 mM and different [L]/[P]. As a control, aLUV data in the absence of peptide are also shown.

In the absence of peptides, no significant changes of scattering intensity were observed during the time course of the experiments (*i.e.* ∼24 hours). This signifies that the produced aLUVs are sufficiently stable for all experiments presently reported. The addition of L18W-PGLa induced an equilibration of lipid distribution across both leaflets with a rate that strongly increased with peptide concentration (Fig. 4). Analysis in terms of eqn (4) yielded flip-flop half-times of *t*_1/2_ ∼ 500 min at [P]/[L] = 1 : 800, which dropped to 14 min at eight times higher peptide concentration (Table 1). Interestingly, LF11-215 led at equally high [P]/[L] to no detectable lipid flip-flop (Table 1; Fig. S5†). Additionally, we studied lipid flip-flop as induced by MG2a and an equimolar mixture of L18W-PGLa and MG2a. MG2a, similar to L18W-PGLa, is supposed to remain membrane-surface aligned in the present conditions, while its equimolar mixture is well-known for its synergistic activity.^14,31,32^

**Table 1:** Flip-flop rates *k*_f_ and flip-flop half-times *t*_1/2_ for mixtures of asymmetric vesicles with L18W-PGLa, MG2a, their equimolar mixture, and LF11-215 at different [P]/[L] ratios

Peptide	[P]/[L]	*k*_f_ × 10^−5^ (s^−1^)	*t*_1/2_ (min)
L18W-PGLa	1 : 100	42 ± 13	14 ± 4
	1 : 400	11 ± 4	52 ± 16
	1 : 800	1.2 ± 0.4	500 ± 200
MG2a	1 : 100	1.4 ± 0.5	420 ± 140
	1 : 200	<0.6	>10^3^
L18W-PGLa:MG2a	1 : 800	0.8 ± 0.3	700 ± 300
LF11-215	1 : 100	<0.6	>10^3^

Our flip-flop analysis showed that MG2a is significantly less potent than L18W-PGLa in translocating lipids (Table 1; Fig. S5†). No detectable lipid flip-flop was found for [P]/[L] = 1 : 200 and rates at doubled MG2a concentration were comparable to L18W-PGLa at [P]/[L] = 1 : 800. Interestingly, the equimolar mixture of L18W-PGLa and MG2a did not exhibit a faster lipid flip-flop at [P]/[L] = 1 : 800 than L18W-PGLa alone. However, the equimolar peptide mixture contains only [P]/[L] = 1 : 1600 of either L18W-PGLa and MG2a. Considering that lipid flip-flop will drop significantly for AMP these concentrations (
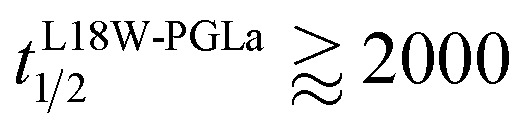
 min; 
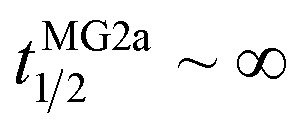
), then suggest that the measured half-time for the peptide mixture is indeed a consequence of L18W-PGLa/MG2a-synergism.

## Discussion

Attempting to gain some deeper understanding of the intricate leakage behaviour of (POPG)^in^/(POPE/POPG)^out^ aLUVs as compared to symmetric LUVs (scrambled and outer leaflet mimics) at [L] = 50 μM and [P] = 125 nM (Fig. 2) we determined peptide partitioning and peptide-induced lipid flip-flop, including LF11-215 and MG2a. All three peptides are able to inhibit bacterial growth, with reported minimum inhibitory concentrations (MICs) of 16 μM (LF11-215), ∼31 μM (L18W-PGLa), and ∼62 μM (MG2a) for *Escherichia coli* K12.^14,24^ Further, while the secondary structure of L18W-MG2a and MG2a once inserted into lipid membranes can be considered as mostly α-helical,^33,34^ LF11-215 due to its short amino acid sequence is expected to be structurally more flexible. In fact, acylated LF11-215, with an octanoyl chain attached to the N-terminal, was reported to form a short α-helical-like turn of five residues in micelles.^35^ Delineating from this study to LF11-215, the structure of LF11-215 can be considered to be a hydrophobic wedge formed by the Phe, Trp and Ile residues, whereas the four Arg residues form a cluster of positive charge.

Thus, it might be expected that LF11-215 partitions differently into lipid membranes than L18W-PGLa; for the presently studied systems and peptide concentrations we found *K*_*x*_(LF11-215) ≥ *K*_*x*_(L18W-PGLa) (Fig. 3). Intriguingly, however, LF11-215 interacted more favorably with OLM than with ScraLUVs, while the opposite partitioning behaviour was found for L18W-PGLa. Yet, differences in partitioning coefficients change significantly with peptide concentration and become negligible for low LF11-215 and L18W-PGLa content. This is a manifestation of the well-known fact that peptide partitioning is a complex non-linear interplay of intermolecular forces beyond mere electrostatic interactions between peptides and lipids. However, our findings might in part be due to the preferred interactions of L18W-PGLa with POPG,^31^ which is enriched in our scrambled vesicles. In turn, insertion of the protonated N-terminal Phe-residue of LF11-215 (next to Trp) into the hydrophobic region should be easier for the less charged OLM whose composition is dominated by POPE.

Strikingly, the observed differences in leakage efficacy of L18W-PGLa are not correlated with its partitioning in membranes. Extrapolating partitioning data to low peptide concentrations, *i.e.* matching leakage and Trp-emission experimental conditions, is most realistically done using a simple propagation of the slopes for *x*_B_, *K*_*x*_ and *f*_B_ at the lowest measured peptide concentration (Fig. 3). A further constraint for the propagation is the requirement 
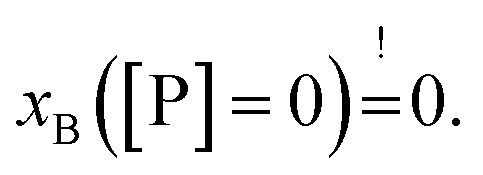
 Using these simple rules suggests a lower partitioning of L18W-PGLa in aLUVs as compared to ScraLUVs or OLM. Note that even if we were to assume a constant *K*_*x*_ for low peptide concentrations (*i.e.*, *K*_*x*_([P] → 0) = *K*_*x*_ ([P] = 0.5 μM)), the actual differences between the *K*_*x*_-values of aLUVs, ScraLUVs and OLM are too small to explain the significantly enhanced peptide-induced dye leakage for aLUVs (Fig. 2). Note also our additional partitioning experiments at higher lipid concentrations, which allowed us to measure at [P]/[L] = 1 : 400, but showed no enhanced peptide association for aLUVs (Fig. S1†). Naturally, we cannot exclude *a priori* a further increase of *K*_*x*_ toward lower [P] for aLUVs. This would imply a sequence of anticooperative → cooperative → anticooperative peptide/peptide or peptide/lipid interactions^27^ with increasing peptide concentration, which appears on the basis of the available data as highly unrealistic. Also the slower onset of leakage of aLUVs as compared to ScraLUVs is unlikely to be an effect of initial anticooperative partitioning interactions, since we found no time-dependence in our Trp-spectroscopy data.

Instead we propose that the intriguing dye-leakage activity of L18W-PGLa in aLUVs is dominated by the elastic/structural response of the bilayer in the presence of the peptide. For example, we have previously demonstrated that insertion and translocation of linear peptides into membranes depend on the elastic energy stress stored within the lipid bilayer.^14^ In particular, POPE, due to its significantly negative intrinsic lipid curvature^36^ and its capability for intermolecular H-bonding^37^ leads to a tightly packed polar/apolar interface and thus an increased free energy barrier for peptide insertion and translocation.

Peptide translocation has been linked to lipid flip-flop even in the absence of the peptide-induced pore formation and, importantly, also to leakage events.^18^ It is therefore interesting to discuss our flip-flop measurements on aLUVs within this framework. Moreover, the high lipid concentrations used for our time-resolved SANS measurements allow us to neglect any effects originating from peptide partitioning,^27^*i.e.* all presently studied AMPs can be assumed to be fully membrane-associated. We found significantly increased lipid flip-flop only for L18W-PGLa (Table 1). Interestingly, LF11-215 did not induce any measurable lipid flip-flop, even at [P]/[L] = 1 : 100, despite its high antibacterial activity (lowest MIC among all presently studied AMPs). Moreover, we previously demonstrated that LF11-215 readily translocates the bacterial envelope of *E. coli*.^24^ Notably, the ability of LF11-215 to induce dye-leakage from vesicles or membrane structural changes has been reported to be rather low as compared to other AMPs.^21,24^ Hence, translocation of peptide through membranes, dye-leakage and lipid flip-flop are not necessarily correlated. Note also that the well-studied AMP buforin II is able to translocate through membranes without inducing lipid flip-flop.^38^ In this case, the Pro-residue was reported to bestow buforin II with a higher structural flexibility to pass through the lipid bilayer. Similarly, LF11-215 is structurally much more adaptable than L18W-PGLa or MG2a, due to its short sequence where only five out of eight residues are able to form an α-helical-like turn. We thus suggest that the structural flexibility enables efficient membrane translocation of LF11-215 without measurable effects on membrane structure or lipid distribution.

On the other hand we argue that lipid flip-flop, leakage and peptide translocation are at least partially coupled in the case of L18W-PGLa and MG2a. Kabelka and Vácha reported from a computational study that the ability for linear peptides to translocate through membranes is connected to the size and distribution of their hydrophobic patches.^39^ In particular, the free energy of membrane insertion was lower for peptides with an increased hydrophobic surface either along the direction of their long axis or at one of their termini. L18W-PGLa and MG2a both have an amidated, *i.e.* non-charged, C-terminus, which inserts first into the membrane upon translocation.^18^ However, the hydrophobic angle of L18W-PGLa, calculated using MPEx,^40^ is significantly larger than that of MG2a (Fig. S6†). Indeed, membrane surface-aligned MG2a was found to be located slightly further away from the bilayer center than L18W-PGLa in POPE/POPG bilayers.^31^ Combination of these pieces of information thus suggests that MG2a is less likely to translocate POPE/POPG bilayers. Moreover, the amidated C-terminus of PGLa was reported to act like a polar brush, shuttling lipids across the bilayer.^18^ A similar mechanism can be expected to apply also to MG2a. Consequently, the significantly lower lipid flip-flop rate in our aLUVs in the presence of MG2a as compared to L18W-PGLa (Table 1) most likely is a corollary of a reduced rate of peptide translocation. The apparently synergistically increased lipid flip-flop for the L18W-PGLa : MG2a equimolar mixture then suggests the facilitated peptide translocation. Although, L18W-PGLa and MG2a were shown to already form heterodimers at low peptide concentrations,^31^ it appears unlikely that these dimers are sufficiently stable to translocate as one entity. Possibly, peptide translocation is assisted by enhanced spontaneous pairwise interactions of the C-termini observed for PGLa alone by MD simulations.^18^

Finally, we return to the significantly increased dye leakage from aLUVs as compared to ScraLUVs and OLM (Fig. 2). Peptides experience along their translocation path not only a free energy barrier upon entering the hydrophobic core of the membrane, but also upon exiting it in the opposing leaflet.^39^ The outer leaflet of our aLUVs is enriched in POPE, while POPG exclusively populates the inner leaflet before the addition of L18W-PGLa. Based on lipid shape-packing arguments we previously reported a significantly lower free energy barrier for bilayers containing cylindrical lipids (such as POPG) as compared to conical lipids (such as POPE).^14^ Hence, translocation of L18W-PGLa in (POPG)^in^/(POPE/POPG)^out^ aLUVs should be energetically easier than in OLM, which densely packs on both sides at the polar/apolar interface. Combined with the reported formation of water bridges and ion leakage during PGLa translocation,^18^ this appears as a plausible scenario to explain the differences in dye-leakage between aLUVs and OLM.

Explaining the different leakage of ScraLUVs and aLUVs using the same arguments is more challenging. Here, the free energy barrier for peptide insertion from the exofacial side is lower than for aLUVs, which possibly relates to the initial more rapid increase of dye leakage. However, at the same time the barrier in ScraLUVs is higher for pushing the peptides out of the hydrocarbon regime in the inner leaflet. This might then account for the final lower leakage levels observed for ScraLUVs. Overall, non-equilibrium contributions to relaxation processes originating from peptides interfering with differential elastic stress stored in compositionally distinct membrane leaflets, plausibly play a significant role, but are difficult to quantify in the absence of a theory.

## Conclusions

Membrane asymmetry adds yet another layer of complexity to antimicrobial peptide activity. Here, the used aLUVs can be seen as first order mimics of inside-out cytoplasmic membranes of Gram-negative bacteria.^19^ While cytoplasmic membrane mimics with ‘correct’ asymmetry and composition lie ahead of some adaptions of cyclodextrin-mediated lipid exchange, the present study still entails some conclusions of physiological relevance. Firstly, and analogously to our previous finding upon including cardiolipin in (symmetric) mimics of cytoplasmic bacterial membranes,^24^ transbilayer lipid asymmetry makes bilayers more vulnerable to AMP attack due to differential tension of the membrane leaflets. However, we cannot exclude that other membrane entities (such as, *e.g.* proteins) help to counterbalance these differences. Secondly, our study corroborates the idea that leakage and antimicrobial activities observed in lipid-only mimics and bacteria are not necessarily correlated. Deep understanding appears to be only within reach, when combining biophysical studies on cells and membrane mimics.^24^

## Author contributions

LM performed experiments, analysed data and wrote the paper. MPKF, GNR and LP performed experiments and analysed data. EFS designed research, analysed data and wrote the paper. KL designed the research. GP designed the research and wrote the paper.

## Conflicts of interest

There are no conflicts to declare.

## Supplementary Material

FD-232-D1FD00039J-s001
