# Supplementary material for: Antimicrobial peptide activity in asymmetric bacterial membrane mimics
Source: Faraday Discuss. 2021 Jul 21;232:435–47. doi: 10.1039/d1fd00039j (PMC8704130; doi:10.1039/d1fd00039j)
Supplement: FD-232-D1FD00039J-s001 [file FD-232-D1FD00039J-s001.pdf]

Electronic Supplementary Material (ESI) for Faraday Discussions.
This journal is © The Royal Society of Chemistry 2021

Supplementary Material

ANTIMICROBIAL PEPTIDE ACTIVITY IN ASYMMETRIC BACTERIAL MEMBRANE MIMICS

Analysis of lipid exchange

Lipid exchange was determined by ultra-performance liquid chromatography-tandem mass spectrometry (UPLC-MS) or gas chromatography (GC) as described in[1].

Ultra-performance liquid chromatography-tandem mass spectrometry

UPLC-MS measurements were performed on an AQUITY-UPLC system (Waters, Manchester, UK) equipped with a BEH-C18-column (2.1 × 150 mm, 1.7 m) (Waters) for sample separation[2]. This system was coupled to a SYNAPTG1 qTOF HD mass spectrometer (Waters) equipped with an electrospray ionization source. MassLynx 4.1 (Waters) was used for data acquisition; lipid analysis was performed with the "Lipid Data Analyzer" software.[?] For UPLC-MS, the mole fraction, χ_i, of a single component can be determined from

$$\chi_i = \frac{P_i}{\sum_i P_i}, \tag{S1}$$

where Pi is the area of the ith lipid peak. This relationship is strictly valid when the lipid peak area fractions vary linearly with mixture composition. UPLC-MS data was calibrated by measuring POPE/POPG mixtures at various molar ratios.

Gas chromatography

To produce fatty acid methyl esters (FAMEs) from lipids, we first evaporated the aqueous solution from our sample using a rotary evaporator at 30 mbar and 40 °C. The dried lipid film was then incubated in a 2.5 vol% H_2SO_4 solution in methanol at 95 °C for 1 h. Once cooled to room temperature, we added 3 ml hexane and 3 ml H_2O, followed by 1 min of vigorous shaking. After letting the emulsion separate for 10 min, we extracted 2.7 ml of the supernatant hexane phase using a pipette and evaporated the solvent under a soft stream of Argon. The remaining film was reconstituted in 70 μl hexane and used for subsequent measurements.

GC measurements were performed using a GC 2010 Plus, Shimadzu, with a split/splitless injector and a SGE BPX70 70% cyanopropyl polysilphenylene-siloxane column (25 m by 0.22 mm ID and 0.25 μm film thickness). We used Helium as a carrier gas at a flow rate of 1.04 ml/min, a linear velocity of 35 cm/s and a purge flow rate of 1 ml/min. Before injection the column was equilibrated at 155 °C for 3 minutes. Then the temperature was ramped up to 180 °C at a rate of 2 °C/min, then to 220°C at a rate of 4 °C/min and finally held at 220°C for 5 min resulting in a 27.5 min total run time. 8 μl of sample was injected into the column at 250 °C using an AOC-20i auto injector. We used a Flame Ionization detector (FID) operating at 260 °C with a flow of 40 ml/min H_2, 400 ml/min compressed air and 30 ml/min He make-up flow. Peak integration was done using the LabSolutions software (Shimadzu).

To evaluate concentration dependence and isotope-specific sensitivity, we performed a concentration series using diplamitoyl phosphatidylcholine (DPPC) and DPPC-d62 (purchased from Avanti Polar Lipids). Concentrations of the stock solutions were measured through an inorganic phosphate assay[3] and converted to equivalent molarities of fatty acids (Tab. S1). The mol-dependent behavior of counts in GC n could be well described by polynomials of second order:

$$\text{mol}(n) = p_2 n^2 + p_1 n + p_0. \tag{S2}$$

Fits and examplary chromatograms are shown in Fig. S3.

Table S1 Results from FAME-GC compositional analysis. The upper section shows integrated counts n from the correspondent peaks to the deuterated and hydrogenated 16:0 FAMEs. The middle section shows fit parameters for each FAME according to eq. (S2). The lower section contains resulting molar amounts and ratios for the sample used in SANS.

	C16:0d	C16:0
Standard (mmol)	n	n
0.0162	53337	55178
0.024	72883	74226
0.048	182893	190370
0.097	316909	330881
0.163	457853	475137
0.242	574354	594897
0.321	669085	697408
0.400	779803	788526
	n	n
Sample 1	654278	382507
Sample 2	729547	424305
Polynomial fits		
p_2 ($\times 10^{-18}$)	5.0208	5.3722
p_1 ($\times 10^{-13}$)	11.4724	6.4426
p_0 ($\times 10^{-7}$)	1.0308	1.4375
	(mmol)	(mmol)
Sample 1	0.300	0.118
Sample 2	0.361	0.138
	(mol%)	(mol%)
Sample 1	71.9	28.1
Sample 2	72.3	27.7
**average**	**72.1**	**27.9**

Supplementary Figures

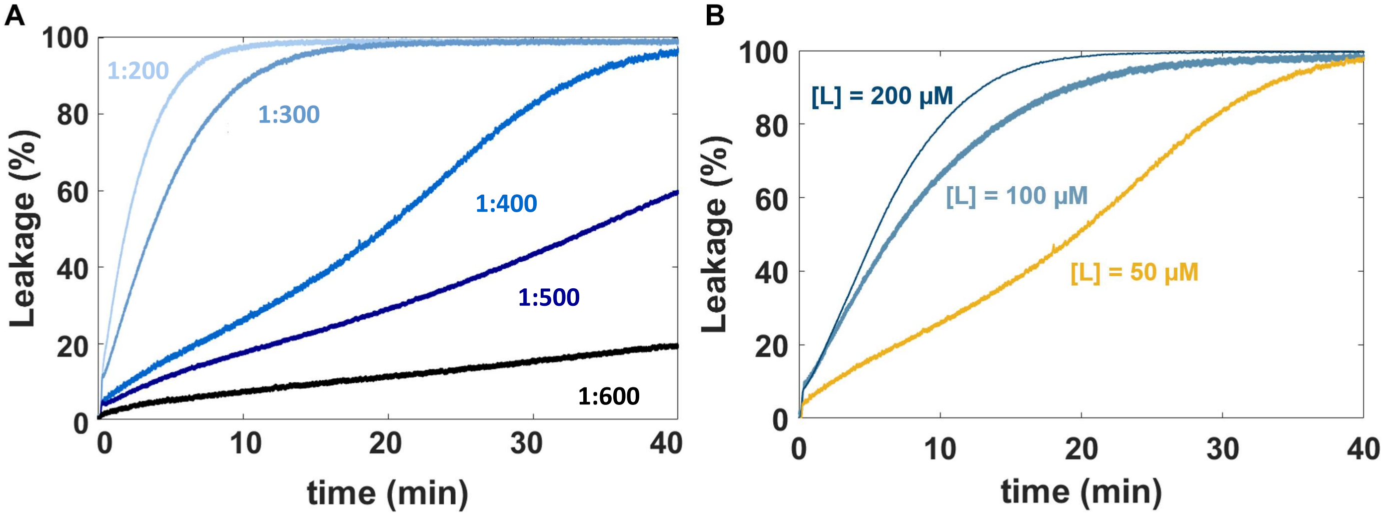

Fig. S1 L18W-PGLa induced dye-leakage in (POPG)^{in}/ (POPE/POPG)^{out} aLUVs for $[L] = 50\,\mu$M at varying peptide concentration (panel A), and $[P]/[L] = 1:400$ and different lipid concentrations (panel B).

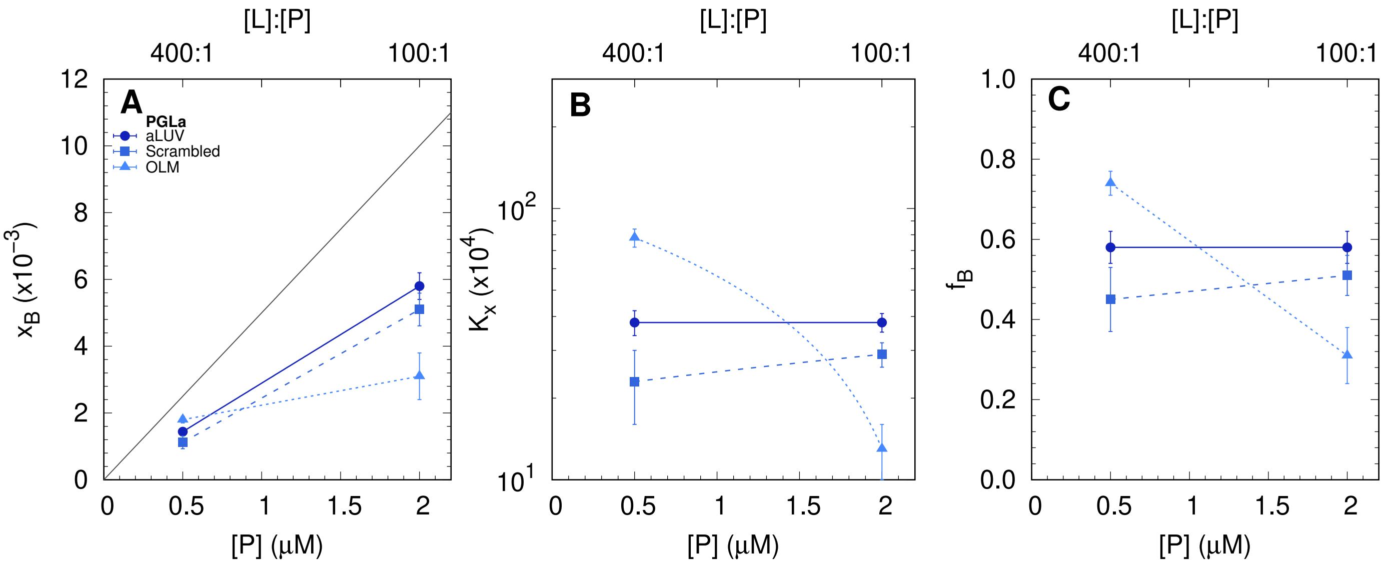

Fig. S2 Mole fraction of membrane-partitioned peptides (A), partitioning coefficient (B) and ratio of partitioned peptides (C) as a function of L18W-PGLa concentration and $[L] = 200\,\mu$M. Data refer to aLUVs (circles) and symmetric scrambled (squares) and outer leaflet mimic (OLM, triangles) systems. The gray line in (A) represents the limit $[P]/[L]$ corresponding to $f_B = 1$; all other lines are guides for the eye.

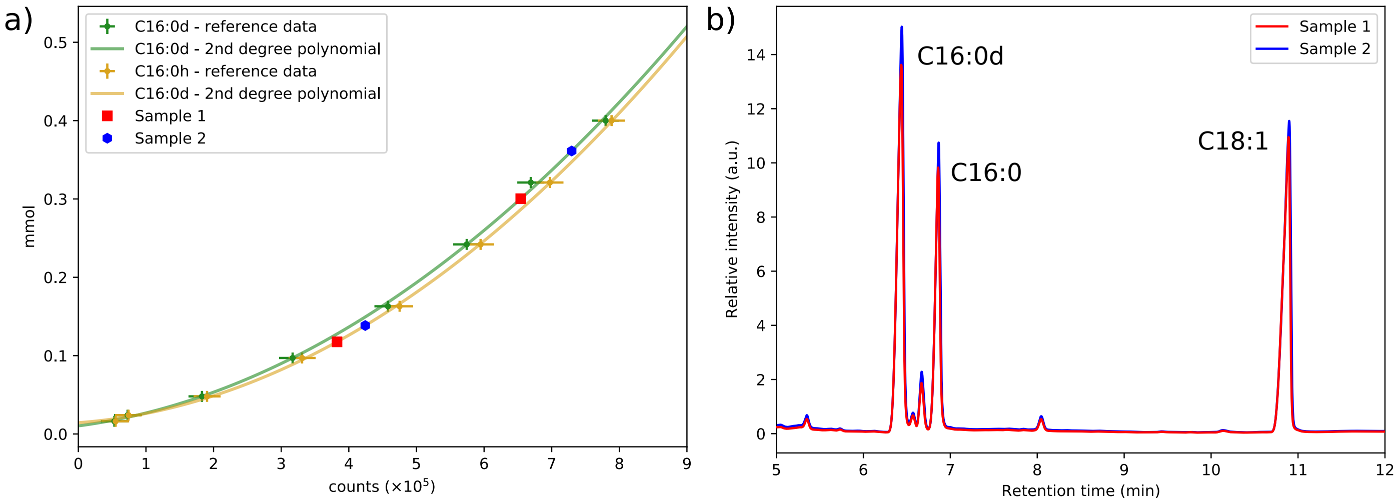

Fig. S3 Compositional analysis of lipid vesicles using GC. a) shows the calibration curve for deuterated and protiated 16:0 FAMEs and second order polynomial fits. Both fatty acids are clearly separated in the chromatograms and highly reproducible as can be seen from technical repeats of the sample used in SANS (b).

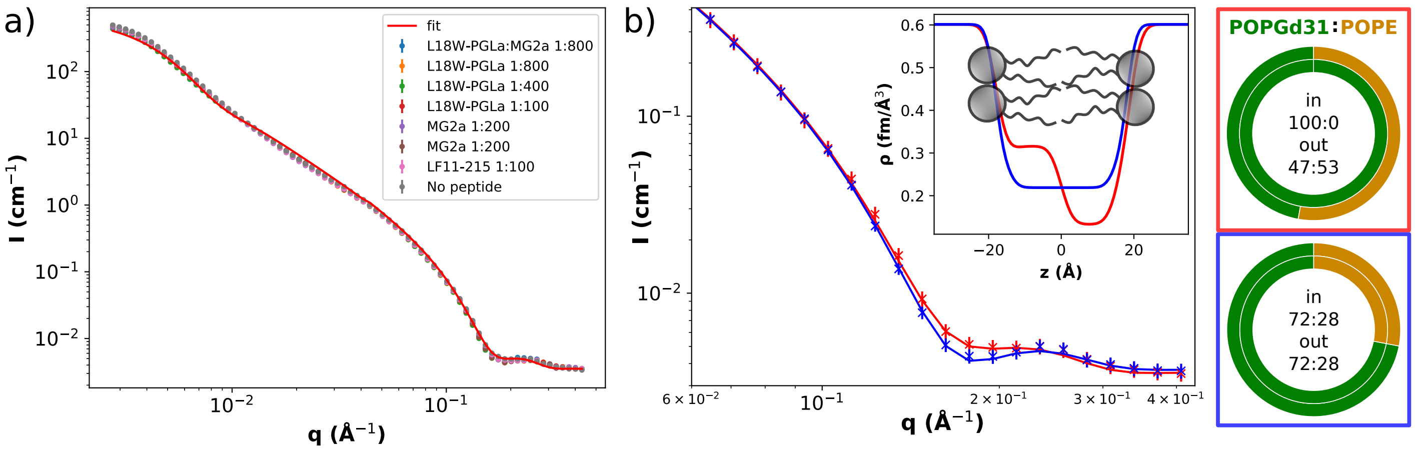

Fig. S4 (a) SANS data of initial asymmetric vesicles without peptide as well as endstates after incubation with peptides for several hours, showing that the overall vesicle structure is unperturbed by the peptides. The solid line is a fit using separated form-factor model of Schultz-distributed vesicles [4] ($s = 0.023$ Å^{-1}, $z = 5.3$). (b) High-q SANS data of asymmetric (red, ×) and scrambled (blue, ×) vesicles. Solid lines correspond to fits, using a 4-slab model [5] for the scattering length density (SLD)-profile, as shown in the inset. SLDs were restricted according to the composition (right side), using previously published values for lipid volumes. [6,7]

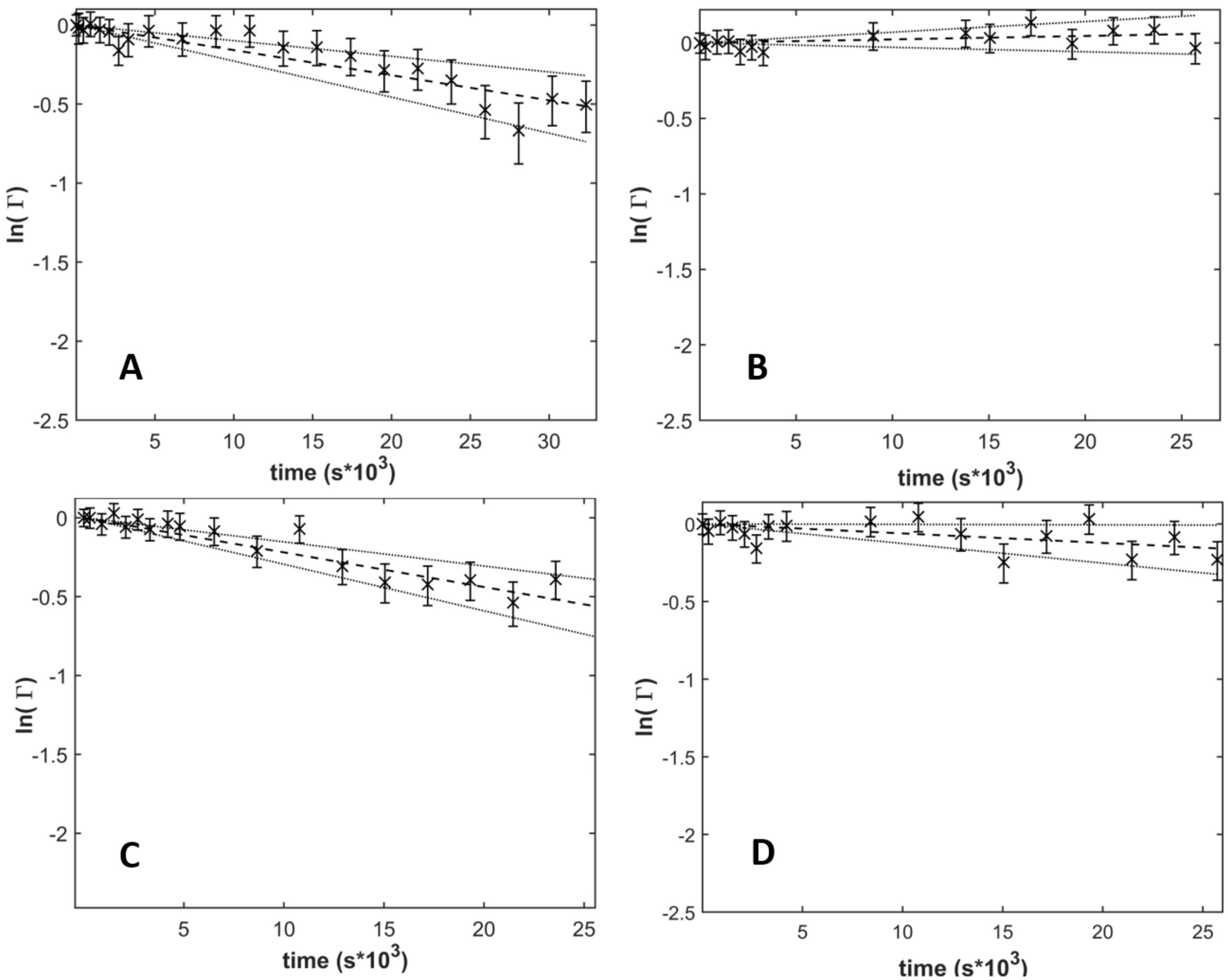

Fig. S5 Kinetics of peptide induced loss of contrast between aLUVs and scrambled vesicles induced by **A)** L18W-PGLa:MG2a ($[P]/[L] = 1:800$), **B)** LF11-215 ($[P]/[L] = 1:100$), **C)** MG2a ($[P]/[L] = 1:100$) and **D)** MG2a ($[P]/[L] = 1:200$). Dashed lines represent best fits to the data and confidence interval.

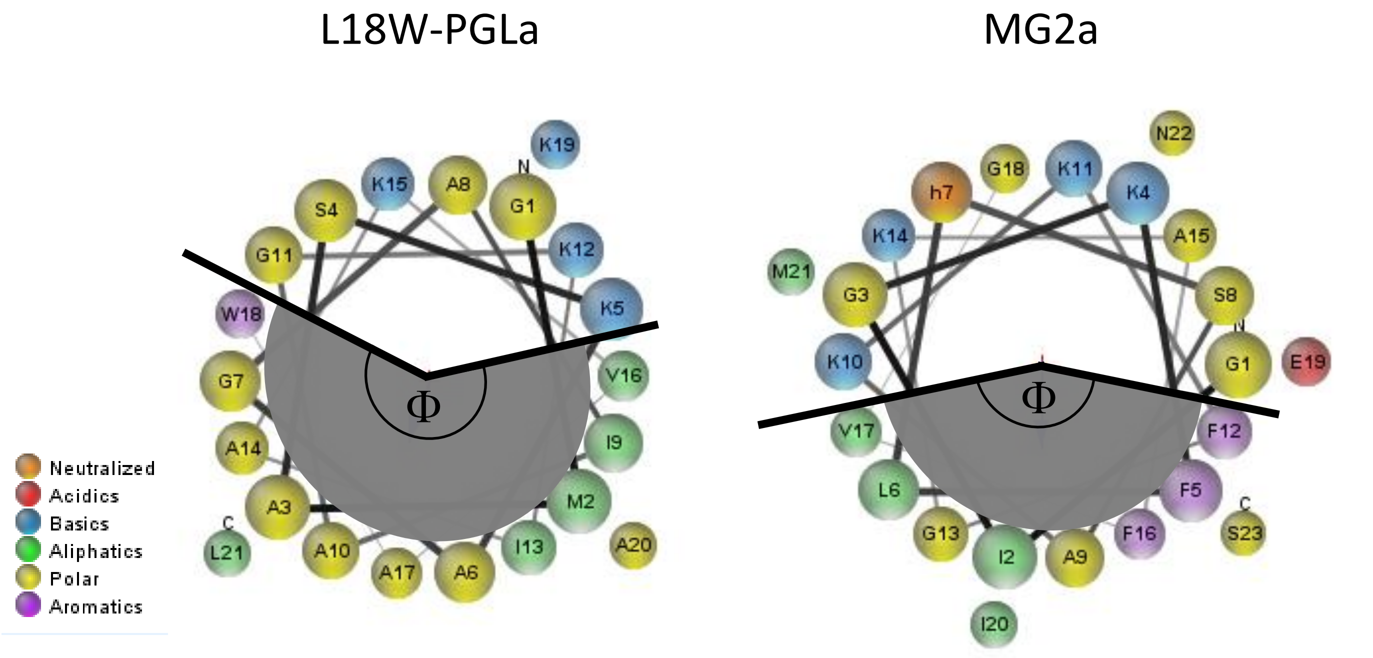

Fig. S6 Helical wheel projections and hydrophobic angles, Φ, of L18W-PGLa and MG2a assuming full helicity using MPEx.[8] For L18W-PGLa $\Phi \sim 215°$ and $\Phi \sim 120°$ for MG2a.

Notes and references

1 M. Doktorova, F. A. Heberle, B. Eicher, R. F. Standaert, J. Katsaras, E. London, G. Pabst and D. Marquardt, 2018, **13**, 2086–2101.

2 O. L. Knittelfelder, B. P. Weberhofer, T. O. Eichmann, S. D. Kohlwein and G. N. Rechberger, *J Chromatogr B Analyt Technol Biomed Life Sci*, 2014, **951-952**, 119–128.

3 P. Kingsley and G. W. Feigenson, *Chem Phys Lipids*, 1979, **24**, 135–147.

4 N. Kučerka, J. Pencer, J. N. Sachs, J. F. Nagle and J. Katsaras, *Langmuir*, 2007, **23**, 1292–1299.

5 B. Eicher, F. A. Heberle, D. Marquardt, G. N. Rechberger, J. Katsaras and G. Pabst, *J Appl Crystallogr*, 2017, **50**, 419–429.

6 J. Pan, D. Marquardt, F. A. Heberle, N. Kučerka and J. Katsaras, *Biochim Biophys Acta*, 2014, **1838**, 2966–2969.

7 N. Kučerka, B. van Oosten, J. Pan, F. A. Heberle, T. A. Harroun and J. Katsaras, *J Phys Chem B*, 2015, **119**, 1947–1956.

8 C. Snider, S. Jayasinghe, K. Hristova and S. H. White, 2009, **18**, 2624–2628.
